# An Overview of Sensory Characterization Techniques: From Classical Descriptive Analysis to the Emergence of Novel Profiling Methods

**DOI:** 10.3390/foods11030255

**Published:** 2022-01-18

**Authors:** Catarina Marques, Elisete Correia, Lia-Tânia Dinis, Alice Vilela

**Affiliations:** 1 Centre for the Research and Technology of Agro-Environmental and Biological Sciences (CITAB), University of Trás-os-Montes and Alto Douro, Apartado 1013, 5001-801 Vila Real, Portugal; catarina.ipsmarques@gmail.com (C.M.); liatdinis@utad.pt (L.-T.D.); 2 Center for Computational and Stochastic Mathematics (CEMAT), Department of Mathematics, University of Trás-os-Montes and Alto Douro, Apartado 1013, 5001-801 Vila Real, Portugal; ecorreia@utad.pt; 3 Chemistry Research Centre (CQ-VR), Department of Biology and Environment, School of Life Science and Environment, University of Trás-os-Montes e Alto Douro, Apartado 1013, 5001-801 Vila Real, Portugal

**Keywords:** discriminative tests, descriptive tests, time-intensity methods, instrumental sensory devices, immersive techniques, sensory data treatment

## Abstract

Sensory science provides objective information about the consumer understanding of a product, the acceptance or rejection of stimuli, and the description of the emotions evoked. It is possible to answer how consumers perceive a product through discriminative and descriptive techniques. However, perception can change over time, and these fluctuations can be measured with time-intensity methods. Instrumental sensory devices and immersive techniques are gaining headway as sensory profiling techniques. The authors of this paper critically review sensory techniques from classical descriptive analysis to the emergence of novel profiling methods. Though research has been done in the creation of new sensory methods and comparison of those methods, little attention has been given to the timeline approach and its advantages and challenges. This study aimed to gather, explain, simplify, and discuss the evolution of sensory techniques.

## 1. Introduction

Sensory science has been successfully used in the beverage industry for centuries. The first reports about sensory perception go back to the ancient Greeks, where Aristotle delineated five of the senses in 350 BC. In the 1600s, Descartes ran some sensory investigations with animals, and the 19th century saw the use of registers about touch, pain, and hot and cold sensations [1]. Only in 1936 was the first attempt for a sensory method published; it was entitled the ‘paired-eating method’ [2]. Then, in 1940, the same author started to approach the selection and training of a panel [3]. Sensory methods became particularly interesting during the 1940s and 1950s, once World War II revealed the importance of nutrition and the development of new products [1].

In those early times, sensory analysis was based on descriptive methods, mainly using natural products as references [4]. Descriptive methods afford objective descriptions of the nature and intensity of sensory characteristics, as well as reliable statistical data. Profiling-based methods and quantitative descriptive analysis were the first descriptive techniques. Over time, descriptive analysis has become more fast-forwarded, flexible, and customized, giving rise to faster techniques. Currently, descriptive analysis is very helpful in the development of products with optimal enjoyment once it is oriented to comprehend and identify the sensory drivers of product enjoyment [5].

According to the literature, by the end of the 1940s and the beginning of the 1950s, several sensory tests emerged. In 1946, Bengtsson and Helm developed the triangle test as a method of select tasters, and in 1947, Dove developed the difference–preference test [6]. According to Rogers [1], discrimination tests had a significant evolution due to the work of Peryam and Swartz in 1950. These investigators defined three tests—the triangle, duo–trio, and dual-standard—for measuring sensory differences [1].

Time-intensity methods were first used in the 1930s when Holway and Hurvich [7] investigated qualitative spatial and temporal patterns induced by a simple saline stimulus, and they recognized that taste intensity changes over time. Dijksterhuis and Piggott [8] reviewed dynamic methods of sensory analysis, realizing that the application of these methods can be beneficial for the study of flavor release. Another interesting review of time-related intensity methods was performed by Lawless and Heyman [9], who provided a major contribution to the acknowledgment of these methods as highly reliable.

Officially sensory panels started around the 1930s, and their use began with the evaluation of products conducted by company sensory experts who applied grading methods [1]. One of the first publications about sensory grading was the investigation of Crocker and Platt [10]. After World War II, due to increasing interest in the development of new food products, the discussion of the best way to recruit a sensory panelist became urgent. Trained panelists would be more accurate and have more experience, despite their vision about a product not always corresponding to consumers’ preferences [1]. A non-trained panel would make a subject assessment about the product and be closer to consumers’ perception [11].

Sensory science has an essential role in the beverage industry. It is used within the framework of product marketing strategies to understand consumers’ preferences and choices [12]; it is also used for product understanding and the creation of new beverages. Nowadays, sensory science has developed numerous consumer methodologies that have boosted the wine industry. Wine sensory analysis emerged in France between the 1950s and the 1970s, intending to validate protected designated origin wines. By that time, descriptive methods using natural products as references were in use [4]. Other beverages such as coffee [13,14,15,16], apple juice [17], iced tea [18], alcoholic cocktails [19], beer [20,21,22,23], and soy-free protein drinks [24] have benefited from the evolution of sensory evaluation methods, improving quality, and creating beverages closer to consumers’ preferences. Additionally, water quality can be improved thanks to sensory studies, making communication easier for consumers when describing water taste and odor and therefore enabling the water industry to better respond to consumers’ feedback [25,26].

This paper is a critical review of sensory techniques from classical descriptive analysis to the emergence of novel profiling methods. Though research has been conducted in the creation and comparison of new sensory methods, little attention has been paid to the advantages and challenges of the timeline approach. This study aimed to gather, explain, simplify, and discuss the evolution of sensory techniques.

## 2. Sensory Descriptive Tests

Qualitative and quantitative descriptive tests are demanding tests in which highly trained panelists are needed to provide the reproducibility of the results. They assume an essential role in the homogenization of “sensory” communication and description of the product through the development of a sensory lexicon. Lexicons develop attributes that qualitatively describe a product and provide quantitative information about the attribute’s intensity [27].

Quantitative descriptive analysis (QDA) is a technique that has been widely used in many studies for quantifying and optimizing sensory attributes [28,29]. Firstly, a sensory panel must be trained to identify and quantify a product’s sensory attributes through appropriate intensity scales so that statistical analysis can be performed [30]. In a previous study [31], Ramirez and co-workers determined the sensory profile of seven watermelon varieties and recruited experienced panelists for descriptive analysis; the first training session consisted of developing a list of attributes, followed by panelists discussing and defining descriptors. After the panelists acquainted themselves with chemical reference standards, they adjusted their attribute perceptions according to those in watermelon samples. Descriptive analysis revealed that the prevalent attributes in watermelons were wateriness, refreshing, crispness, sweetness, mealiness, freshness, ripe, and melon. This technique was also applied to determine the profile of wines [32]. Recent studies have combined QDA with other methodologies and innovative tools such as big data mining [33].

As a variation of QDA, free choice profiling (FCP) is differentiated by the omission of the training phase, which turns make technique into rapid and less time-consuming than QDA; for this to happen, the panel must be familiar with the product category. Assessors choose attributes, and they are free to use as many terms as they want if they systematically use them to characterize the product. Assessors must decide attributes and meanings before categorization. After products are presented one by one to the assessor, the perceived intensity of the attribute is evaluated through a scale. Since expertise is missing in FCP, a significant challenge is a lack of accuracy. The individual profile data are analyzed by a multidimensional technique called Generalized Procrustes analysis [34,35], a methodology is used to characterize and distinguish products with different properties [36].

Flash profiling (FP) has its roots in FCP; it consists of an evaluation based on assessors’ attributes [35], and it can be implemented with an untrained panel. FP was the first method that emphasized rapidity, and it allows for the understanding of the sensory positioning of products. Although FP does not put out terms, assessors should prioritize descriptive terms rather than hedonic terms in general [34]. Furthermore, this method leads assessors to look for differences between samples [37], and it has proven to be a suitable discriminative sensory method for beverages such as coffee [13] and wine [35].

Liu and co-workers [35] proposed a modified version of FP that became an efficient screening of sensory properties in the case of wine. This alternative involved the napping methodology with subsequent attributes as the word-creation step and a restricted number of terms in the product ranking. This modified version seemed to be more effective for discrimination [35]. New FP approaches are being developed for different kinds of beverage and food products [38,39].

There have been some investigations into the combination of projective mapping (PM) and ultra-flash profiling. These methods in symbiosis significantly contribute to identifying similarities and differences between samples [40,41,42].

PM is a fast-sensory technique that asks a panel to rank products based on their similarities and differences in a two-dimensional plan, creating a graphical representation. This technique enables the description of products through their similarities and differences, as well as the clustering samples [27,43]. It can be performed with different kinds of panelists (experienced panelists, trained panelists, naïve consumers, or individuals who are employed in the wine industry), allowing for comparisons of results to study consumers’ perception [42,44,45,46]. However, when time, resources, or samples are limited, an experienced panelist may be the best option [45]. Other authors have proven that PM can be successfully implemented in a wide variety of beverages categories, e.g., herbal tea infusions [47], chocolate-flavored milk [48], wines [41,49], and soy-free protein drinks [24]. PM was also proved to be an effective technique to explore food-beverage pairings [50], and recent studies have shown that PM is being used as part of new approaches, namely the affective approach [51], intensity approach [41], hedonic frame [52], and upgrades such as polarized projective mapping [49]. The affective approach substantiates product categorization based on consumers’ choices or preferences [51]. The intensity approach investigated by Wilson and co-workers [41] assesses the relation of different levels of intensity of two thiols (3-mercaptohexan-1-ol (3 MH) and 3-mercaptohexyl acetate (3 MHA)) in various matrices. Kim and co-workers [52] compared a hedonic frame of projective mapping that asked consumers to group samples based on similar reasons for liking or disliking those products, with a sensory frame of projective mapping that asked consumers to group based on sensory resemblances. After mapping the results, they used ultra-flash profiling in both sensory projective mapping and hedonic projective mapping, where assessors could freely describe the sensory attributes of the groups they had categorized. Polarized projective mapping has been used as a solution in studies with larger sample sets or multiple complex categories such as wine. This solution incorporates the terminology “poles” from polarized sensory positioning [53]. Polarized projective mapping uses the location of poles previously established on the panelist’s map; panelists are asked to create a bi-dimensional map, as in projective mapping, but “free-moving” samples are proposed for the panelists to set out around the poles that represent solid references [49].

The check-all-that-apply (CATA) methodology is based on the principles of pre-selected sentences or statements where assessors, even without any previous training, can check the ones that apply to that product [54]. It is a fast and straightforward method that is easy to merge with affective measurements, such as hedonic tests [55]. Additionally, CATA can be used with an untrained panel, and there is evidence that CATA results in better accuracy of results with training [56]. However, CATA term-citation frequency is strongly linked to direct rates of perceived intensity, though this does not mean that intensity can be assessed by CATA questions [57]. CATA questions have already been applied to the sensory characterization of a wide range of products of different complexity [58]; regarding beverages, we can highlight its use for apple juice, iced tea [18], wine [59], and milk chocolate [60]. Recent studies have presented some approach variations such as check-if-apply as a solution to water quality judgment, where the focus was undesirable attributes. One of the CATA method requirements is that the product has many desirable and undesirable terms. As such, this novel approach suggests a list of descriptors and asks consumers to choose the few of them that apply [26]. Rate-all-that-apply (RATA) is another alternative to the CATA method where consumers not only check but also rate the intensity of all attributes they find in the assessed product [61]. Furthermore, emerging approaches assume a more dynamic direction, e.g., temporal check-all-that-apply (TCATA) [62].

Open-ended questions are helpful to understand consumers’ perceptions. Initially, they were used for comprehending consumers’ reasons for liking a product. Nowadays, they comprise a valuable tool to understand consumers’ perceptions and which attributes lead to the preference of the product. This method gives an assessor complete freedom of expression, but it is a time-consuming method. Redundancy, ambiguity, and the extension of terms are some of the challenges of open-ended questions [63]. Deneulin and Bavaud [64] presented a textual data treatment from open-ended questions about minerality perception in wine without a tasting phase. In this research, they considered quantitative aspects without considering semantic or linguistic aspects.

In the preferred attribute elicitation (PAE) method, assessors determine several descriptive terms for products; after agreeing on these attributes, they rank their liking intensity of those attributes [65]. This novel sensory method can be used with an untrained panel in just a single session. Defining the most relevant attributes for consumers allows industries to design new food products that meet consumers’ preferences [23]. Discrete choice experiments (DCEs) and best-worst scaling (BWS) are two typical elicitation methods. In DCEs, participants select between two or more possibilities. BWS can work via three different approaches: object cases, profile cases, and multi-profile cases. In profile cases, respondents choose the best and worst alternatives from a list of dimension levels or items [66]. A recent investigation by Campigotto and co-workers [67] proposed CLEO, a preference elicitation algorithm that allows for the suggestion of complex configurable objects represented by discrete and continuous attributes and the constraints defined over them. Some studies have suggested the use of PAE and CATA [68] and PAE and TCATA combined [65].

Polarized sensory positioning (PSP) is based on a reference (pole), and samples are compared with those poles. There are no attributes that allow for a faster sensory characterization and more straightforward data analysis in this method. PSP can be classified into two types with different kinds of evaluation: polarized sensory positioning based on the degree of different scales and triadic polarized sensory positioning. In the former, the scale ranges from “exactly the same” to “totally different”. In the last one, assessors are asked to have poles in consideration and display which sample is more similar and which one is more different [69].

Introduced by Lawless and co-workers [70], sorting (also known as a free sorting task or free multiple sorting) is used to investigate perceptual models of odor perception. This method is an excellent option for untrained assessors [71]; however, an expert sensory panel is crucial for accurate sensory characterization [33,72,73]. It can also be reproduced with healthy older adults [74] due to its quick and straightforward applicability, which is why it has become such a popular method. In just one session, products can be randomly and simultaneously presented in different sequences. Tasters are invited to sensory evaluate and sort products into groups with perceived similitudes. Then, they give minor descriptors to characterize each of those groups [27].

Table 1 illustrates the development of sensory descriptive tests; it identifies the type of evaluation, the origin of the sensory lexicon, the statistical data treatment used, advantages, limitations, and variations of each test.

## 3. Sensory Discriminative Tests

Discriminative tests determine whether samples are similar or there is some difference between samples and, if so, which one is different. According to the complexity of the product, the type of discriminative test is chosen, and it is implemented based on several parameters such as the replacement of an ingredient in the product, the installation of new equipment, or deviations from usual protocol during production [85].

One of the most popular discriminative tests is the triangle test. Firstly, the triangle test was used for the quality assessment of whiskeys and beers, and then its use spread to other beverage and food products [86]. In Debela and co-workers’ investigation [87], 94.4% of a sensory panel was capable of distinguishing between *Coffea Arabica* honey and *Vernonia amygdalina* honey. In this test, three samples are displayed to assessors at the same time. Two of these assessed samples are the same, and one of them is different. Samples are presented at random, making combinations such as AAB, ABA, BAA, BBA, BAB, and ABB [86]. After coding random samples with three digits, assessors identify the odd one out, assessing samples from left to right. Statistically, assessors are likely to get it right 1 out of 3 times or 33.3% [85]. A triangle test can be used to identify a difference between two products, market trends, and the impact of a change in ingredients, packaging, processing, handling, or storage conditions; it is also a helpful tool in the recruitment process of a tasting panel [86]. Accuracy and assessment time in triangle tests do not increase when considering monetary incentives; however, if assessors like the product, these aspects can be impacted [88].

Unlike the triangle test, the tetrad test focus on similarities between samples. Four samples, in which two are from one group and two are from a second group, are displayed to assessors [85]. The tetrad test can be very useful to understand how consumers perceive changes in the production process or even changes in some ingredients [89]. This test is reviewed as more powerful than the triangle test; despite the probability of correctly answering the same in both tests (1/3), the tetrad test has a higher statistical significance. Therefore, it can be seen as a more efficient and accurate test [85,89], as well as being considered a forced-choice test [89].

The duo–trio test was created as an alternative to the triangle test because it is easier to perform than the triangle test [90]. In this test, assessors are presented with three coded samples, in which one of which is the reference. Assessors may identify the most similar sample to the reference. This test can be used to evaluate how significant sensory differences are between samples [91].

Duo–trio tests are classified into two designs: constant-reference mode and balanced-reference mode. In the former, the reference is constant during the entire test. It is chosen when assessors are more familiar with one of the samples and when there is a limited quantity of a sample [90]. In the balanced reference mode, both samples are randomly presented as references. New versions with variations of the place where the reference may remain balanced are gaining prominence; the reference can be presented first or in the middle. Even dual reference duo–trio tests have been suggested in the literature, with the first and last places, the first and middle places [91], or comparisons between pairs of distances [92]. For a better comparison of multiple pairs, A-Not A with a reminder (A-Not AR) and 2-AFC with a reminder (2-AFCR) can also be used [93].

Similar to the duo–trio test but with two served reference samples is a dual-standard test. This test may assume several possible combinations, namely, reference A and reference B (pause), coded A and B samples; reference A and reference B (pause), coded B and A; or the positions of the references and services that are switched [94].

Following the structure of the duo–trio test but reversed is ABX. First, assessors are given two control samples and a treated sample, and then they are asked to match the “X” sample to one of the references [94].

The A-Not A test is another discriminative method that consists of presenting reference A and other samples to the assessor, who must then choose whether the other assessed samples are similar or not to the A sample [91].

As discriminative tests such as the triangle or duo–trio tests can lead assessors to sensory fatigue with strong flavors and complex products, paired comparison tests are a suitable solution because they are simpler and more intuitive. In a paired comparison, assessors are asked to compare two samples without considering the intensity of perception. Paired comparison tests can be classified as simple difference tests or directional paired comparison tests (or 2-alternative forced-choice (2-AFC) tests); usually, they are implemented with two samples, but they are also possible with multiple samples (multiple paired comparison test). Their application can be based on forced-choice (FC), which means that assessors must choose one of the two samples, or non-forced-choice, where assessors have the alternative “no difference,” which means both samples seem similar to them [95]. To increase forced-choice power and detect small and directional changes of stimuli, some paired versions of FC tests have been emerging [96]. One is known as an alternate forced-choice (AFC), which can be based on the triangle test becoming 3-AFC or a paired comparison test becoming 2-AFC [91].

One variation is the four-interval, two-alternative forced-choice (4I2AFC), which is a paired version of the 2-AFC where the two alternatives are AB and BA pairs, a stronger stimulus or signal is considered, and the weaker stimulus or noise is B. In 4I2AFC, assessors are asked to choose the pair (AB) with decreasing stimuli change [97].

Table 2 illustrates the development of sensory discriminative tests; it identifies the type of evaluation, statistical data treatment, advantages, limitations, and variations of each test.

## 4. Sensory Hedonic Tests

Hedonic methods are characterized by their ability to measure the subjective individual response of consumers’ preferences, acceptance, liking, or perception of a product’s benefits [27]. There have been some investigations into optimization methods such as just-about right (JAR) scaling and Ideal Profile Method (IPM) [104]. The bimodal JAR scales point out sensory terms that interfere the most with product acceptance. The “just–right” level of a sensory term is represented by a midpoint in the scale. The points at the ends are extremes, such as not smooth enough or too smooth [105]. This scale is frequently used in product development with an untrained panel or consumers, and it allows for the measurement of JAR attributes on enjoyment [106]. In IPM, the intensity and ideal intensity of attributes for each product is rated by assessors. This method is key in the early development of the sensory qualities of existing products [107]. In addition to hedonic measurements, a forthcoming scale is known as the degree of satisfaction-difference (DOSD) was created to validate consumers’ preferences. This scale considers the consumer’s context and internal evaluative criteria before product assessment [108].

Research on consumers’ understanding and emotional response towards beverages has been gaining interest [16,22,109,110]. Even new methods are emerging, such as relative preference mapping (RLM), which provides information about wine styles that are liked and innovative based on projective mapping to measure consumers’ preferences [111].

**Figure 1:**
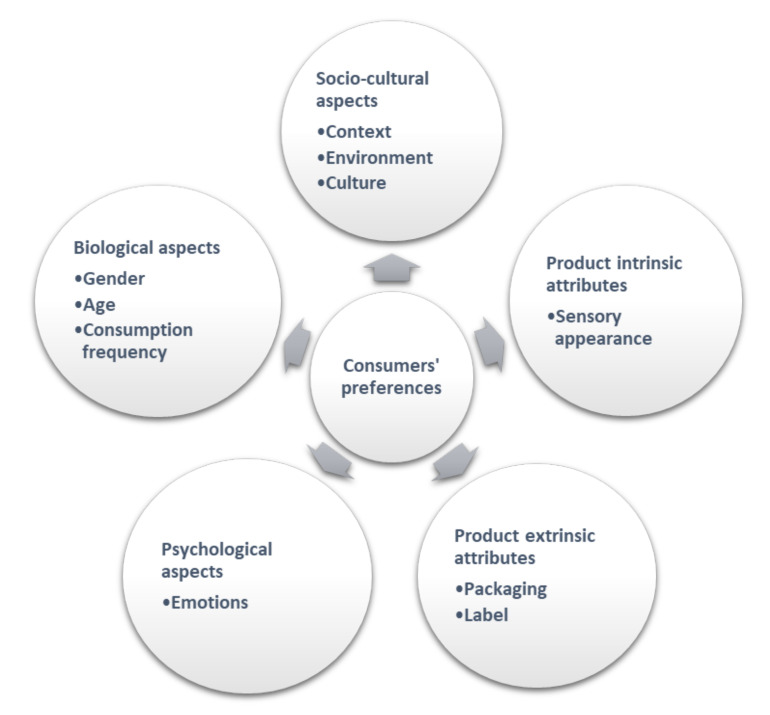
Factors that influence consumers’ preferences. Adapted from [22,112,113,114].

Many factors, including biological, psychological, and socio-cultural, may influence consumers’ preferences and choices (Figure 1) [22]. Gender, age, consumption frequency, education, and income are just a few examples of those many variables that affect consumers’ preferences and choices [112]. In addition, product-intrinsic attributes such as sensory appearance, product-extrinsic attributes such as label or packaging [113,114], and contextual and environmental influences may have clear effects on hedonic tasting [22].

Context and the consumer’s mood may also affect the evoked emotions, creating an association between elicited emotions and the willingness to pay more for the product [115]. Furthermore, cross-cultural studies have received particular attention [16], but there are no standardized differences between cultures’ responses [116].

Emotions influence product experience and product consumption, and for this reason, they are essential in consumer behavior [117]. Wine consumption is associated with pleasure by wine consumers, and their emotions impact wine consumption experiences [118]. Functional and emotional associations can motivate consumption [20]. Emotions elicited by consumption can also provide additional information about consumers’ personalities [119]. Recent research has used individuals’ factors to segment consumers to understand their preferences [111]. Segmentation based on psychographics and behavior was studied by Danne et al. [120], who investigated the impact of context on wine consumer segments’ enjoyment and emotions while consuming wines in different environments.

In sensory and consumer science, cross-cultural research has become stronger. The main linguistic differences across cultures are sensory terms, emotional terms, and the interpretation of scale anchors. American consumers use a more extensive range of nine points for hedonic scales than Asian consumers [116].

## 5. Temporal Tests

The way consumers perceive a product is strongly linked to their expectations, which can be based on their enjoyment or even their satiety. A temporal driver approach can be completely appropriated to trigger those expectations in consumers with diverse eating preferences and behaviors during the tasting process [121]. Over the last few years, temporal dynamics in the sensory assessment of beverages have been widely investigated [19,122,123,124,125,126,127] because the sensory analysis is a very complex and dynamic process that floats and evolves.

Time-intensity (TI) methods consider the intensity of stimuli over time, and they perform incredibly well in the analyses of sensations, namely the evolution of mouthfeel and flavor release [128]. TI can be classified as a dual attribute time-intensity (DATI) method if assessed with two stimuli or a multiple attribute time-intensity (MATI) method if assessed with multiple stimuli. The main goal of these methodologies is to define a pattern of the evolution of a specific sensory characteristic. Although time–intensity is perfect for contrasting products with different temporal characteristics [27], it requires extra training and more repetitions to achieve reliable results; thus, the TI Reliability Index was suggested to explore intra-individual variation in the same panel [122]. In cases of products with shorter consumption times such as chewing gum, the continuous time-intensity (CTI) method has performed well, constantly recording assessors’ perceptions. Furthermore, other intensity methods such as temporal dominance of sensations (TDS) evaluate various attributes during the assessment of a sample; CTI provides deeper and customized data regarding the perceived intensity of an attribute and its variations over time [128].

TDS is better than temporal dominance methods due to its ability to consecutively record several sensory attributes over time, identifying one specific attribute as “dominant” [37]. TDS is more effective regarding temporal differences than TI, though it does not mention why an attribute is dominant; it is also a less time-consuming technique. Scales in TI are not equal and do not allow for the comparison of attributes [129]. Nevertheless, there are other temporal dominance approaches. One of them is temporal liking (TL), which is used to collect scores and perceive variations of the acceptability of a product over time [124]. TL can be alternated with TDS [130], recognizing temporal drivers of liking by TDS [131] or performing temporal liking simultaneously with temporal dominance of sensations in several intakes [132]. temporal dominance of emotions (TDE), where sensory attributes are replaced by emotions, was recently proposed as an extension of TDS [133]. This technique is widely used to understand factors that lead consumers to buy a product, such as packaging color [134]. Recently, some authors suggested new pathways to apply TDE, dynamically recording facial expressions for assessing food-elicited emotions over time [135,136] or applying video advertisements of a product [133]. There are periods during tasting where there is no dominant attribute, and that can create noise in data. In the hold-down procedure, assessors hold down the attribute button when it is perceived as dominant and release it when it is no longer dominant [137].

Another popular temporal method is temporal check-all-that-apply (TCATA). TCATA is a dynamic method for describing several sensory features of a product and its development over time. Based on the CATA method in TCATA, assessors are asked to check all attributes that apply to the product in evaluation in addition to recording the evolution of sensory changes in products [138]. One of the applications of this method is in the measurement of wine complexity [139]. Moreover, further investigation has been made based on a combination of two or more temporal methods. For example, to characterize wines from different varieties, TDS and TCATA have been used, and it was concluded that in combination with phenolic composition, these methods are helpful in the detection of the time of bitterness perception [140].

Although TDS and TCATA are frequently used for sensory evaluation during consumption, they present some struggles because both rely on a predefined and shortlist of attributes. To overcome that limitation, Mahieu and co-workers suggested free comment attack evolution finish (FC AEF), where assessors describe a product through free comment descriptions during periods, namely attack, evolution, and finish [141].

A new method called projective categorization was created to predict wine aging potential, giving assessors a visual tool to assess the projected development of a wine’s quality over time over different dynamics. In a study, three curves in an orthonormal coordinate system were given to the assessors; these curves corresponded to three aging potentials (high potential, medium potential, and low potential) for Champagne base wines. Assessors were asked to place the tasted wine on one of these three curves. Assessors based their choices on the temporal notion (with the abscissa axis) and the qualitative notion (with the ordinate axis) following the aging potential evaluation. This method allowed for the accurate distinction of wines with different aging aptitudes [142].

Table 3 illustrates the development of temporal tests; it identifies the type of evaluation, data acquisition method, statistical data treatment, advantages, limitations, and variations of each test.

## 6. Instrumental Sensory Devices and Immersive Techniques

**Figure 2:**
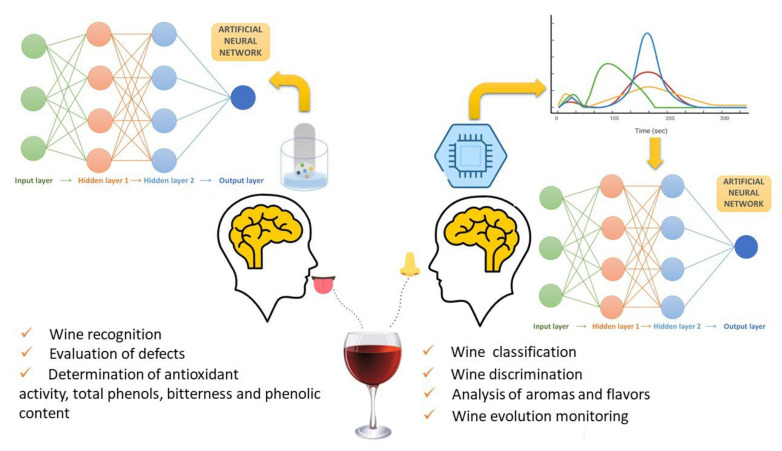
Working principle of an e-tongue and e-nose system. Adapted from [150,156].

The use of instrumental sensory devices such as e-nose and e-tongue and immersive techniques has been growing in beverages analysis [149,150,151], such as wine properties detection [152,153,154,155,156,157,158,159] (Figure 2).

The electronic nose (e-nose) was developed to imitate the olfactory system of humans [154]. The use of this device involves the transition of an aroma into electrical signals through several chemical sensors. The hardware learns how to identify different patterns and classify the wine aroma among a class of aromas that previously have been taught [159]. For each chemical compound, the device can have up to 40 sensors, and to receive and process data, the equipment must contain the following components: a multisensory array where the assessed sample is delivered, an artificial neural network (ANN) that detects the sample, and a computing system with digital pattern-recognition algorithms and reference-library databases (Figure 2). There are several signal transduction mechanisms of the e-nose, and in all of them, the collection of data and the classification of the analyte will be better when the number of sensors in a cross-reactive sensor array (CRSA) is higher [27]. The e-nose has several attractive features due to its quick analysis of headspace, ability to qualitatively represent an aroma, and cost-efficiency of [159]; however, it also has some weaknesses, such as some irrelevant noise of major compounds for aroma, the presence of sensor drift or poisoning, and the presence of ambiguous information because of the sensor’s responses [160]. To overcome these challenges, one of the future pathways will be hybrid devices, as present research is starting to develop such systems [161]. In the wine industry, e-nose devices are used to detect and control wine quality in real-time [152,153] and to distinguish and identify wines with different properties [162].

Following the same logic, an electronic tongue (e-tongue) was created to mimic human gustative receptors. Created in the 1990s, the e-tongue involves “a multichannel electrode with transducers composed of lipid membranes immobilized with a polymer” [160]. In situations of automatic process control, poisonous or extreme condition samples, or cost efficiency, the e-tongue can be a great alternative to a human expert panel [150]. E-tongues are designed with electrochemical sensors such as voltammetric, potentiometric, amperometric, impedimetric, and conductimetric or biosensors such as optical or enzymatic sensors [160]. In the case of electrochemical sensors, current research has merged FTIR (Fourier-transform infrared spectroscopy) with voltammetric e-tongues based on SPE (Solid-phase extraction) in red wines. This methodology allowed for the rapid evaluation of several parameters in a single experiment [155]. For biosensors, the authors of [163] combined tyrosinase and glucose oxidase enzymes and polypyrrole or polypyrrole/AuNP (Polypyrrole-Coated Gold Nanoparticles) composites to analyze and discriminate musts and wines. Bioelectronic tongues contribute general information about products and data about specific compounds due to their biosensors [156]. Hybrid sensors have been further investigated. For simultaneous aqueous and gaseous analyte investigation, the combination of e-tongues with e-noses [164] and the fusion of e-noses, e-tongues, and computer vision have been proposed for the measurement of color and surface characteristics [160].

Immersive approaches are gaining much interest in sensory sciences. A compelling methodology to understand consumers’ behavior and preferences and to improve product design is part of the virtual reality context, in which it is possible to change the visual features of food and beverage products without changing their composition. Within the reality–virtuality continuum, there are scales from the real environment to augmented reality and from augmented virtuality to the virtual environment [165]. These dynamic tools have opened the potential for new immersive and interactive systems. Traditionally, virtual reality has been implemented with the use of a stereoscopic head-mounted display (HMD). However, nowadays, it has expanded to an entirely immersive experience with visual and auditory control and tactile and kinesthetic features using haptic gloves, full-body haptic suits, and motion-tracked controllers. Augmented reality is a more recent concept, where virtual and real objects simultaneously coexist to create an illusion. This technology started with overlaying visual imagery onto the real world, but there have been attempts to create audio-driven augmented reality glasses [166]. Virtual reality can be applied in various areas, such as the sensory evaluation of food [165] and beverages [150], consumers’ preferences, emotions, and behaviors [167,168]. Jiang et al. [118] studied the impact of wine flavors and context through an immersive environment on the consumer perception of green and floral flavors; this study revealed that although the immersive context did not affect the flavor perception, floral wine elicited more positive emotions than green wine.

Gaming is an emerging method in sensory science, with positive outcomes in health prevention and promotion [169]; education in learning factories [170], sensory education, and tasting activities (specifically in children’s novel vegetable intake to promote a diversity of food choice [171]); and the acceptance of products, such as encouraging children to taste fruits and vegetables [172]. It also has had determinant roles in medicine when students or professionals are learning procedures/protocols [173,174]. Another application of serious games is in children with visual impairments, where they can be used to improve the children’s psychosocial well-being [175].

## 7. Sensory Data Treatment

Over the years, statistical techniques have been forced to overcome once sensory science demands by increasing their specificity and accuracy.

Currently, one of the most applied techniques for descriptive and discriminative tests is principal component analysis (PCA) (Table 1 and Table 2). PCA is a multivariate pattern recognition method that can be applied to characterize a sensory profile and compare products [176]. Recent studies have considered other applications such as the acquisition of information about d-prime values across sensory attributes [103], the analysis of the impact of treatments on a product’s shelf life, the detection of correlations between studied responses [177], and the contribution of product positioning with correct approaches or strategies [30]. More comprehensive statistical techniques have emerged in recent literature, including LASSO-PCA (least absolute shrinkage and selection operator, - principal component analysis) comprehensive evaluation for matcha sensory quality [178].

Regarding the free sorting task, statistical data treatment can be performed by employing means of correspondence and cluster analyses [80], as well as DISTATIS (analysis of multiple distance matrices) [71]; however, it is essential to have statistical expertise. As an alternative analysis tool, sorting backbone analysis introduces a simple network to identify groups of significantly alike products and create precise visual results such as graphs [63]. For big data treatment, new tools have been presented, e.g., data mining [33] and natural language processing [179]. Silva and co-workers suggested a new sensory approach combined with a text-mining tool to create a sensory lexicon and profile of monovarietal apple juices [17].

Data analysis for hedonic tests consists of binomial tests or when presented with more than two products, a nonparametric test such as Kruskal–Wallis. A nine-point hedonic scale is applied to assess acceptability. These kinds of ordinal data are usually analyzed by interval-scale data and paired t-test or ANOVA [103].

For sensory data analysis, there are other statistical techniques such as multivariate analysis of variance (MANOVA) [180,181,182], cluster analysis [183], correspondence analysis [14], multidimensional scaling analysis [182], hierarchical cluster analysis [176], partial least squares regression [184], multiple linear regression [185], and GPA [34].

Another important milestone reached in recent years was the use of data analysis with non-parametric MANOVA [179] and categorical principal components analysis (CATPCA). CATPCA explores correlations between variables (ordinal, nominal, and numeric) and explains the common dimensions of the variables. It can be used for variable selection and dimension reduction when categorical variables (also ordinal) are involved [184].

For sensory descriptive analysis, many statistical data treatments can be applied, including regression analysis, factor analysis, confirmatory factor analysis, path analysis, and discriminant analysis. According to Vilela and co-workers [185], structural equation modeling (SEM) proved to be an adequate model for the description of monovarietal wines. SEM is suitable for reducing perceived variables, such as sensory terms, by exploring the covariances between the observed variables [186].

## 8. Comparison of Methodologies

As researchers seek novel sensory methodologies, there is a need to improve the efficiency of such methodologies. It is crucial to compare the consistency of each method and its applicability to obtain successful results.

Previous studies concerning the comparison of descriptive tests support the idea that they are effective in characterizing samples; however, their limitations can determine the selection of the method [68,187,188]. Mahieu and co-workers explored the stability of free-comment and CATA in two consumer studies on red wines and milk chocolates; in this study, free-comment proved to be slightly more stable than CATA [60]. For launching new products into the market, a study by Denize and co-workers revealed that PSP, CATA, PM, or Napping could be efficient when applied to probiotic chocolate-flavored milk [189]. Moreover, in the specific case of wine flavor assessment, Liu and co-workers tested Napping and FP and found that Napping was a better method for enhancing qualitative sample differences while FP allowed for the more accurate product mapping regarding quantitative differences between model wines [38]. In another study on red wine assessment with descriptive analysis, FCP, FP, and a modified version of FP were used. This study showed that although descriptive analysis contributed more precise information, it was too time-consuming compared to the modified version of FP, which was slightly more stable when they increased the number of assessors [35].

Usually, discriminative tests can be applied in all situations when the attribute is unknown; however, some tests have a particular advantage when applied in a specific situation. In the case of samples with strong flavors, two-sample presentation tests are preferable to diminish sensory fatigue [190]. Triangle and tetrad tests were compared by Burns and co-workers, who concluded that the statistical advantages of the tetrad test are not always found in practice [85].

Previous studies have compared rapid with dynamic sensory methods [58,65,191,192]. A consumer sensory profile using PAE and TCATA revealed that PAE allowed for a complete characterization of samples, and TCATA indicated how the attributes changed and evolved through time [65]. Another study comparing CATA with TCATA claimed that both methods presented the same information, but TCATA gave information about the evolution over time [58].

There is no unanimity about the best method for beverages; it always depends on the complexity and specificity of the beverage, as well as the kind of results that are desired. However, it is possible to say that methods based on attribute assessment are more discriminative in terms of detecting small differences than methods that consider similarities between samples.

Considering cross-cultural studies, methodologies such as projective mapping and sorting that conduct a holistic assessment of similarities and differences should be advantageous because there is no need to translate sensory terms before their application [116].

## 9. Topics for Future Research

This review has revealed that sensory science is full-steam ahead in creating and optimizing sensory methodologies. It is possible to believe that methods will be increasingly more powerful and fewer assessors will be needed in the future.

Sensory data treatment will see advancements, with expansions of its specificity, rigor, and ability to analyze sets of multivariate data and big datasets. Technology is one of the promoters of this statistical adjustment, and its use will escalate and lead to more home sensory evaluations of beverages.

Finally, immersive contexts and cognitive psychological contribution will reinforce sensory assessment as a holistic experience and generate an exceptional understanding of beverages.

## 10. Conclusions

Descriptive tests are crucial for the homogenization of the sensory lexicon. They also require a lot of effort, as highly trained panelists need to deliver reproducibility of the results.

Discriminative tests have been applied according to the complexity of products, and they can identify similarities and differences between samples. As traditional discriminative tests lead assessors to have sensory fatigue, paired comparison tests can be used as a perfect solution; much simpler and straightforward variations of paired comparison and forced-choice have been introduced.

For the subjective assessment of consumers’ perception, preferences, acceptance, and enjoyment, hedonic methods such as just-about right scaling and the Ideal Profile Method have been optimized, and some new methods such as relative preference mapping have been emerging.

A temporal approach can be appropriated to trigger consumers’ expectations during the tasting process. To explore intra-individual variation in the same panel, a time-intensity Reliability Index was suggested as a variation of the time-intensity method. Other proposed variations include the temporal dominance of sensations, continuous-time–intensity, temporal dominance of emotions, and temporal check-all-that-apply.

The use of instrumental sensory devices such as e-noses, e-tongues, and immersive techniques has been growing. Gaming is also an emerging method in sensory science, with positive outcomes in many fields.

Statistical techniques are extremely important for sensory data treatment. The most common techniques are principal components analysis and analysis of variance. Statistical techniques for sensory data analysis include multivariate analysis of variance, cluster analysis, correspondence analysis, multidimensional scaling analysis, hierarchical cluster analysis, partial least squares regression, multiple linear regression, Generalized Procrustes analysis, categorical principal components analysis, and structural equation modeling.

## Figures and Tables

**Table 1:** Classification of sensory descriptive tests.

Test	Type of Evaluation	Lexicon	Statistical Analysis	Advantages	Limitations	Variations	Ref.
QDA ^1^	After the training phase, assessors develop qualitative attributes and provide quantitative data about the attribute’s intensity	Provided by a trained panel	ANOVA ^2^; PCA ^3^	Allows for the determination of product profiles	Time-consuming and requires a training phase	FCP ^4^	[28,30,75]
FCP ^4^	Assessors develop qualitative attributes and provide quantitative data about attribute’s intensity without the training phase	Elicited by assessors or a predetermined list	GPA ^5^	Rapid and less time-consuming	Lack of accuracy	FP ^6^	[34,76]
OEQ ^7^	Verbal description of samples	Elicited by the assessors	MFA ^8^; CA ^9^; Chi-square test	Complete freedom of expression	Time-consuming, Has redundancy, has ambiguity, and requires the extension of terms	Textual data treatment from open-ended questions	[77,78]
Sorting; FS ^10^; FMS ^11^	Classification of samples based on their similarities and differences	Elicited by the assessors orprovided by the researcher	DISTATIS; CA ^9^; MDS ^12^	A fast and straightforward method that can be used in a single session	All samples should be presented simultaneously	SBA ^13^; Q-sort method; CS ^14^; FS ^15^; FMS ^16^; HS ^17^	[70,79,80]
PM ^18^; Napping	Generating samples on a two-dimensional map according to their similarities and differences	Elicited by the assessors	MFA ^8^	Description through product similarities and differences, as well as the clustering samples	All samples should be presented simultaneously; difficult to understand for naïve consumers	Affective approach; intensity approach; hedonic frame; PPM ^19^	[40,43,51,52]
FP ^20^	Ranking of samples on a set of selected attributes	Elicited by the assessors	GPA ^5^; CVA ^21^; PCA ^3^; MFA ^8^	Rapid	Two sessions are required. All samples should be presented simultaneously	Modified FP ^20^ with napping Pivot Profile	[81,82]
PAE ^22^	Ranking of attributes according to assessors’ liking intensity of those attributes	Elicited by the assessors	GPA ^5^; HCA ^23^ PANOVA ^24^	Only one session is required	A round-table discussion is necessary; all samples should be presented simultaneously	Discrete choice experiments; best-worst scaling; CLEO ^25^	[23,65,67]
CATA ^26^	Pre-selected terms, where assessors choose the ones that apply to the product	Provided by the researcher	Cochran Q test; MFA ^8^; Chi-square test	A fast and straightforward method that is easy to merge with affective measurements, such as hedonic tests	The design of the term list could influence the answers; not recommended for evaluating very similar samples	Check-if-apply; RATA ^27^; TCATA ^28^	[26,83,84]
PSP ^29^	Evaluation of global differences between samples and a set of fixed references	Elicited by the assessors	ANOVA ^2^; PCA ^3^; MDS ^14^; MFA ^8^; GPA ^5^; CA ^9^	A fast and straightforward method	Stable and readily available references are needed; selection of references couldstrongly affect the results	PSP ^28^ based on the degree of different scales and triadic PSP ^29^	[25,69]

Legend: ^1^. Quantitative descriptive analysis; ^2^. analysis of variance; ^3^. principal component analysis; ^4^. free-choice profiling; ^5^. Generalized Procrustes analysis; ^6^. flash profiling; ^7^. open-ended questions; ^8^. multiple factor analysis; ^9^. correspondence analysis; ^10^. free sorting; ^11^. free multiple sorting; ^12^. multidimensional scaling; ^13^. sorting backbone analysis; ^14^. constrained sorting; ^15^. fixed-sorting; ^16^. free multiple sorting; ^17^. Hierarchical sorting; ^18^. projective mapping; ^19^. polarized projective mapping; ^20^. flash profiling; ^21^. canonical variate analysis; ^22^. preferred attribute elicitation; ^23^. hierarchical clustering analysis; ^24^. Procrustes analysis of variance; ^25^. combinatorial utility function joint learning and optimization; ^26^. check-all-that-apply; ^27^. rate-all-that-apply; ^28^. temporal check-all-that-apply; ^29^. polarized sensory positioning.

**Table 2:** Classification of sensory discriminative tests.

Test	Type of Evaluation	Statistical Analysis	Advantages	Limitations	Variations	Ref.
Triangle test	Identification of a different sample from a set of three samples.	Mixed model logistic analysis; mixed ANOVA ^1^; Tukey’s test	Does not require specification of the nature of the difference	Lack of accuracy; ineffectiveness and sensory fatigue; requires large sample sizes to be effective	Tetrad test; duo–trio test	[85,87]
Tetrad test	Group similar samples from a set of four samples.	Hypothesis testing	Fewer assessors can be used to recover the same confidence in the result	Sensory fatigue		[89,98]
Duo–trio test	Three samples are displayed; one of them is the reference. Identification of the most similar sample regarding the reference.	Hypothesis testing	Easier performance in complex or hard-to-evaluate products; the ability to evaluate how significant sensory differences are between samples	Sensory fatigue; large assessor groups need to be used to increase confidence in the data; low statistical power	CRM ^2^; BRM ^3^; A-Not AR ^4^; 2-AFCR ^5^; different positions of references; ABX	[90,91,92,99]
ABX test	Two control samples and a treated sample are presented to assessors, and they are asked to match the “X” sample to one of the references.	Hypothesis testing	Participants do not need anyprior knowledge of the samples; assessment of fewer products	No guidance over an attribute to focus on; less sensitive test; relies on the assessors’ memory		[100,101]
A Not-A test	Reference A and other samples are presented to assessors, and they must decide whether the other samples assessed are similar to the A sample.	Chi-squared test; Thurstonian distance	Single presentation test; usable with high carryover effect samples	Less recommended when assessors are untrained and/or with no experience with the products		[91,102]
Paired Comparison	Compares two samples without concerning the intensity of perception.	PCA ^6^; Friedman test; Bradley–Terry model	Simple and intuitive task; sensitiveness to differences between stimuli	Time-consuming. Low statistical power	Simple difference tests or directional paired comparison tests (or 2-alternative forced-choice tests); multiple paired comparison test; FC ^7^	[91,95,103]
FC ^7^	Assessors must choose one of the two samples.	ANOVA ^1^	Simple task	A tendency for “noise” in the datasets	Triangle test; AFC ^8^; can be based on the triangle test becoming 3-AFC or paired comparison test becoming 2-AFC; 4I2AFC ^9^	[95,98]

Legend: ^1^. Analysis of variance; ^2^. constant-reference mode; ^3^. balanced-reference mode; ^4^. A-Not A with a reminder; ^5^. 2-AFC with a reminder; ^6^. principal component analysis; ^7^. forced-choice; ^8.^ duo–trio test alternate forced-choice; ^9^. four-interval two-alternative forced-choice.

**Table 3:** Classification of sensory temporal tests.

Test	Type of Evaluation	Data Acquisition	Statistical Analysis	Advantages	Limitations	Variations	Ref.
TI ^1^	Tracks the evolution of the intensity of sensory attributes over time		ANOVA ^2^; PCA ^3^	Quantification of the continuous perceptual changes that occur in a specific attribute over time	Time-consuming when used on several attributes	DTI ^4^; DATI ^5^; MATI ^6^	[19,143]
TDS ^7^	Records several sensory attributes consecutively over time, identifying one specific attribute as “dominant”	Compusense ^8^; EyeQuestion^® 9^; Fizz ^10^; TimeSens ^11^	PCA ^3^; ANOVA ^4^	Effective regarding temporal differences; Less time consuming; Simpler task foruntrained consumers	Not so adapted to trained panels	TDL ^12^; TDE ^13^; HDTDSE ^14^	[144,145]
TCATA ^15^	Assessors are asked to check all attributes that apply to the product in evaluation in addition to recording the evolution of sensory changes in products	Compusense at-hand 5.6 ^16^	Randomization Tests; Cochran’s Q Test; McNemar’s Test; binomial test	Continuous selection and deselection of attributes based on applicability of the attribute to describe a sample	More complicated for the consumer		[139,145,146]
TL ^17^	Collects scores and perceives variations of the acceptability of a product over time	TimeSens^®^	ANOVA ^4^; LSD ^18^	Easier performance in complex or hard-to-evaluate products The ability to evaluate how significant sensory differences are between samples	Sensory fatigue; large assessor groups need to be used to increase confidence in the data; low statistical power	TDE ^13^	[124,147]
TDE ^13^	Records several emotions consecutively over time, identifying one specific emotion as “dominant”	TimeSens 1.0 ^19^; FaceReader™; An adapted version of EsSense Profile^®^	ANOVA ^4^; AHC ^20^; MDA ^21^	Allows for the evaluation of food evoked emotions as motivators for food choices	Risk of simulated emotions	HDTDSE ^14^; TDFE ^22^	[133,136,148]
HDTDSE ^14^	Assessors hold down the attribute button when it is perceived as dominant and release it when it is no longer dominant	TimeSens ^23^	ANOVA ^4^; CVA ^24^; MANOVA ^25^	Allows for subjects to report indecisive behavior	Does not overcome classic temporal dominance in terms of sensitivity and discrimination ability		[137]
FCAEF ^26^	Assessors describe a product through free comment descriptions during periods, namely attack, evolution, and finish	TimeSens© ^27^; IRaMuTeQ©	Bootstrap test; Fisher’s exact tests; Chi-square test; CA ^28^	Description of the temporal evolution with complete freedom of expression	Time-consuming, Redundancy, ambiguity, and requires an extension of terms		[141]
PC ^29^	Assessors place samples on one of three curves		A statistical method developed by [146]	Quantifies three dimensions simultaneously	Requires a large number of assessors		[142]

Legend: ^1^. Time-intensity; ^2^. analysis of variance; ^3^. principal component analysis; ^4^. discrete time-intensity; ^5^. dual attribute time-intensity; ^6^. multiple attribute time-intensity; ^7^. temporal dominance of sensations; ^8^. Compusense (Guelph, Ontario); ^9^. EyeQuestion^®^ (Elst, the Netherlands); ^10^. Fizz (Biosystèmes, Couternon, France); ^11^. TimeSens (Tsi, SAS, Dijon, France); ^12^. temporal drivers of liking; ^13^. temporal dominance of emotions; ^14^. hold-down temporal dominance of sensations and emotions; ^15^. temporal check-all-that-apply; ^16^. Compusense at-hand 5.6 (Compusense Inc., Guelph, Ontario, Canada); ^17^. temporal liking; ^18^. least significant difference; ^19^. TimeSens 1.0 (INRAE Dijon, France); ^20^. agglomerative hierarchical cluster; ^21^. multidimensional alignment; ^22^. temporal dominance of facial emotions; ^23^. TimeSens (version 1.1.601.0, ChemoSens, Dijon, France); ^24^. canonical variate analysis; ^25^. multivariate analysis of variance; ^26^. free comment attack evolution finish; ^27^. TimeSens© software 2.0 (INRAE, Dijon, France); ^28^. correspondence analysis; ^29^. projective categorization.

## Data Availability

Not applicable.
